# Analysis of transcription factors expressed at the anterior mouse limb bud

**DOI:** 10.1371/journal.pone.0175673

**Published:** 2017-05-03

**Authors:** Shigetoshi Yokoyama, Soichi Furukawa, Shoya Kitada, Masaki Mori, Takeshi Saito, Koichi Kawakami, Juan Carlos Izpisua Belmonte, Yasuhiko Kawakami, Yoshiaki Ito, Tempei Sato, Hiroshi Asahara

**Affiliations:** 1 Department of Systems Biomedicine, National Institute of Child Health and Development, Setagaya, Tokyo, Japan; 2 Department of Systems BioMedicine, Tokyo Medical and Dental University, Bunkyo, Tokyo, Japan; 3 Division of Molecular and Developmental Biology, National Institute of Genetics, and Department of Genetics, SOKENDAI (The Graduate University for Advanced Studies), Mishima, Shizuoka, Japan; 4 Gene Expression Laboratory, Salk Institute for Biological Studies, La Jolla, California, United States of America; 5 Department of Experimental Medicine, The Scripps Research Institute, La Jolla, California, United States of America; Laboratoire de Biologie du Développement de Villefranche-sur-Mer, FRANCE

## Abstract

Limb bud patterning, outgrowth, and differentiation are precisely regulated in a spatio-temporal manner through integrated networks of transcription factors, signaling molecules, and downstream genes. However, the exact mechanisms that orchestrate morphogenesis of the limb remain to be elucidated. Previously, we have established EMBRYS, a whole-mount *in situ* hybridization database of transcription factors. Based on the findings from EMBRYS, we focused our expression pattern analysis on a selection of transcription factor genes that exhibit spatially localized and temporally dynamic expression patterns with respect to the anterior-posterior axis in the E9.5–E11.5 limb bud. Among these genes, *Irx3* showed a posteriorly expanded expression domain in *Shh*^*-/-*^ limb buds and an anteriorly reduced expression domain in *Gli3*^*-/-*^ limb buds, suggesting their importance in anterior-posterior patterning. To assess the stepwise EMBRYS-based screening system for anterior regulators, we generated *Irx3* transgenic mice in which *Irx3* was expressed in the entire limb mesenchyme under the *Prrx1* regulatory element. The *Irx3* gain-of-function model displayed complex phenotypes in the autopods, including digit loss, radial flexion, and fusion of the metacarpal bones, suggesting that *Irx3* may contribute to the regulation of limb patterning, especially in the autopods. Our results demonstrate that gene expression analysis based on EMBRYS could contribute to the identification of genes that play a role in patterning of the limb mesenchyme.

## Introduction

Developmental morphogenesis advances through a set of rules and interweaving gene interactions that regulate specification, proliferation, and differentiation [1, 2]. The morphogenesis of appendages, such as limb buds, progresses through well-established mechanisms involving axis formation, patterning, outgrowth, and differentiation. From this viewpoint, the limb bud acts as an excellent developmental model for studying the formation of the vertebrate body plan. However, the precise mechanism orchestrating the integrated steps of morphogenesis during development still remain to be elucidated [3].

The tetrapod limb consists of three anatomical structures: the stylopod (humerus, femur), zeugopod (radius/ulna, tibia/fibula), and autopod (hand, foot). Limb bud outgrowth arises from the lateral plate mesoderm at a defined somite level of the body trunk [4–6] and continues to grow along the three axes: the proximodistal (PD), anteroposterior (AP), and dorsoventral (DV) axis. In the chick embryo, axis formation begins at a signaling center called the “organizer”. The organizer is a group of cells that specifies the identity of nascent mesenchymal cells along the AP or PD axis, such as the apical ectodermal ridge (AER) at the tip of the limb bud [7–9] or the zone of polarizing activity (ZPA) at the posterior region of the limb bud, respectively [10, 11]. Sonic hedgehog (SHH) in the ZPA [12] was identified as the morphogen responsible for controlling the AP polarity of the limb bud. However, this observation could not explain all the published research findings from genetic experiments and called for further analysis to postulate a theory that not only complied with experimental findings but also integrated novel viewpoints [1, 2].

Increasing genetic approaches using mouse models aided the understanding of addition key factors or mechanisms involved in the patterning of the limb bud. During AP patterning of the limb, the mutual antagonism between *Hand2*, which function upstream of *Shh* expression in the ZPA, and *Gli3*, which restricts *Shh* expression to the posterior mesoderm controls the pre-patterning of the AP axis [13]. Thus, in the limb bud, concentration and temporal gradients of SHH exposure controls the digit pattern [14, 15].

High-throughput biochemical experiments implied regulation of many downstream targets by *Shh* and *Gli3* in the correct spatiotemporal manner [16], indicating the critical role of this regulatory network in integrative morphogenesis [1, 2]. However, the precise timing, location, and function of these regulatory factors that form the complicated regulatory network leading to the developmental processes, are still unknown.

Studying these transcriptional regulators provide greater insight into developmental and differential processes [6, 17, 18]. Accordingly, we had established a whole-mount *in situ* hybridization (WISH) database named EMBRYS that displays the spatiotemporal expression of transcription-associated genes (e.g.: transcription factors or cofactors) of mouse embryos at embryonic days (E) 9.5, 10.5, and 11.5 [19]. To understand the transcriptional hierarchy involved in the developmental mechanism that regulate limb bud patterning and digit formation, 1520 transcription factors or cofactors were categorized according to the 3-D distribution of transcripts displayed in EMBRYS, into developmental candidates for each part of the embryonic body as previously shown [19]. Among these, 691 candidate genes expressed in E9.5–11.5 limb buds expected to be involved in limb bud development were identified. These were first screened for transcription-associated factors with characteristic spatial localizations and temporal expression dynamics that suggested a role in AP patterning in the developing mouse limb bud. The factors were then screened using WISH to identify the downstream target genes of *Shh* or *Gli3* that mainly regulated the AP patterning in the developing limb bud. Furthermore, *Shh*-deficient embryos were used for WISH analysis to narrow down on the candidates that might play a role in the anterior patterning of the limb bud beyond SHH, thus identifying *Irx3*, *Asb4*, *Lhx2*, *Nr4a2*, and *Hhex*. The expression of these candidates in *Gli3*-deficient limb buds was analyzed. Notably, each candidate displayed distinct and strikingly different expression pattern changes in *Shh-* and *Gli3-* deficient limb buds.

Markedly, *Irx3* showed significant expression change in *Shh-* and *Gli3-* deficient limb buds. To assess the EMBRYS-based stepwise selection model of candidate factors for developmental processes *in vivo*, a transgenic mouse that overexpressed *Irx3* under the control of *Prrx1* promoter (*Prrx1-Irx3* mice) was generated. The *Prrx1-Irx3* mice exhibited anomalies in the thumb and digits with an almost complete penetration, signifying the lack of AP polarity in their autopods.

Taken together, the present study demonstrates the application of our EMBRYS-based screening strategy for the selection, identification, and characterization of key developmental factors expressed at the anterior end of the mouse limb bud.

## Materials and methods

### Whole-mount *in situ* hybridization

**Fig 1:**
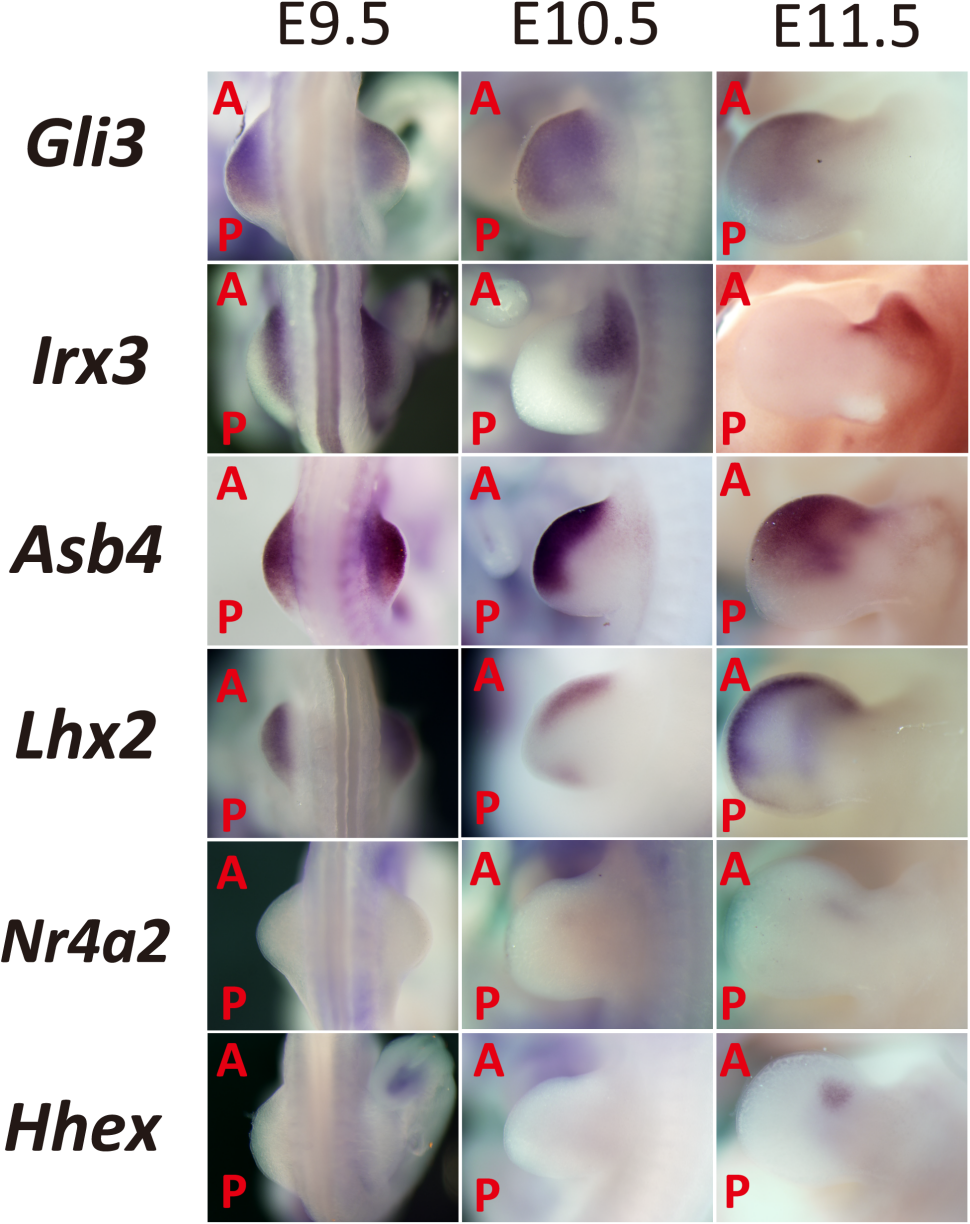
Expression pattern of anterior-localized genes in the limb bud based on EMBRYS. Candidate transcription-associated factors involved in limb bud patterning were selected from EMBRYS and classified according to their anterior expression patterns. (A: Anterior; P: Posterior).

**Fig 2:**
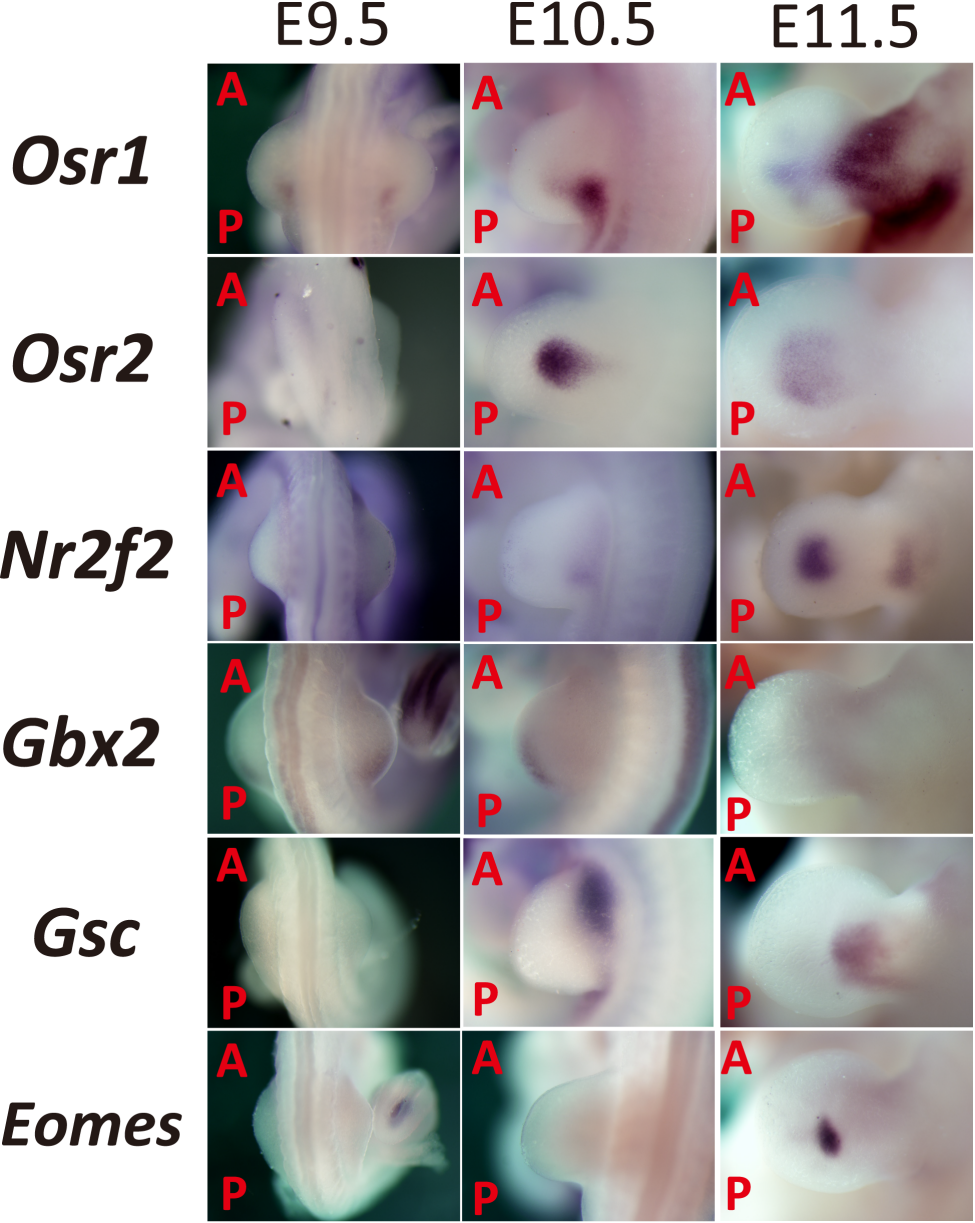
Expression pattern of candidate’s specifying the central part of the mouse limb bud based on EMBRYS. Candidate transcription-associated factors involved in limb bud patterning were selected from EMBRYS and classified according to their expression pattern, particularly those showing significant localization in the limb bud mesenchyme. (A: Anterior; P: Posterior).

**Fig 3:**
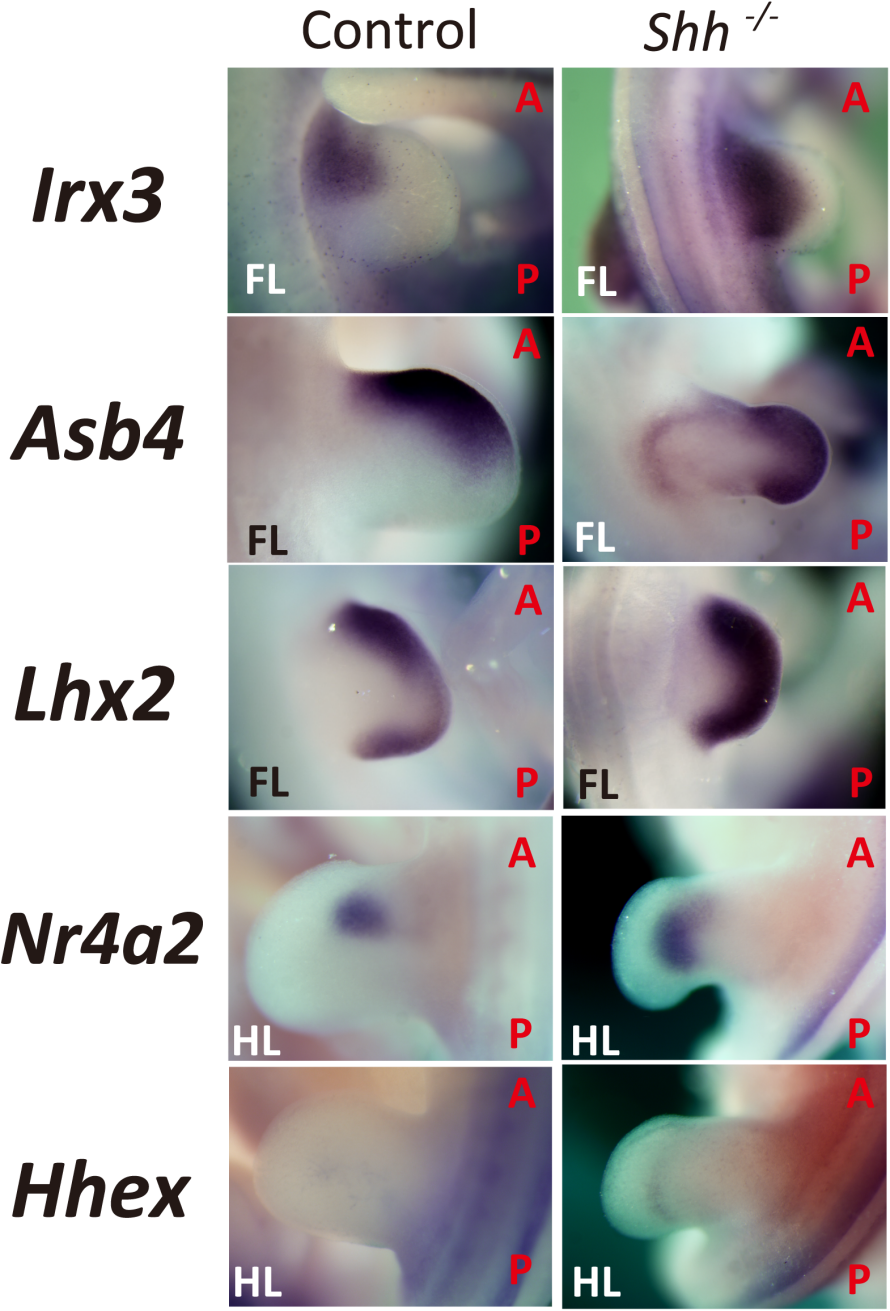
Expression pattern of candidates that are anterior regulators but expanded posteriorly in *Shh-KO* mouse limb buds. Whole-mount *in situ* analysis was performed upon limbs of *Shh-KO* embryos (E10.5–11.5). Disruption of *Shh* resulted in posteriorly expanded expression of candidates in the developing limb bud (Control: WT or *Shh* +/-; FL: Forelimb; HL: Hind limb; A: Anterior; P: Posterior).

**Fig 4:**
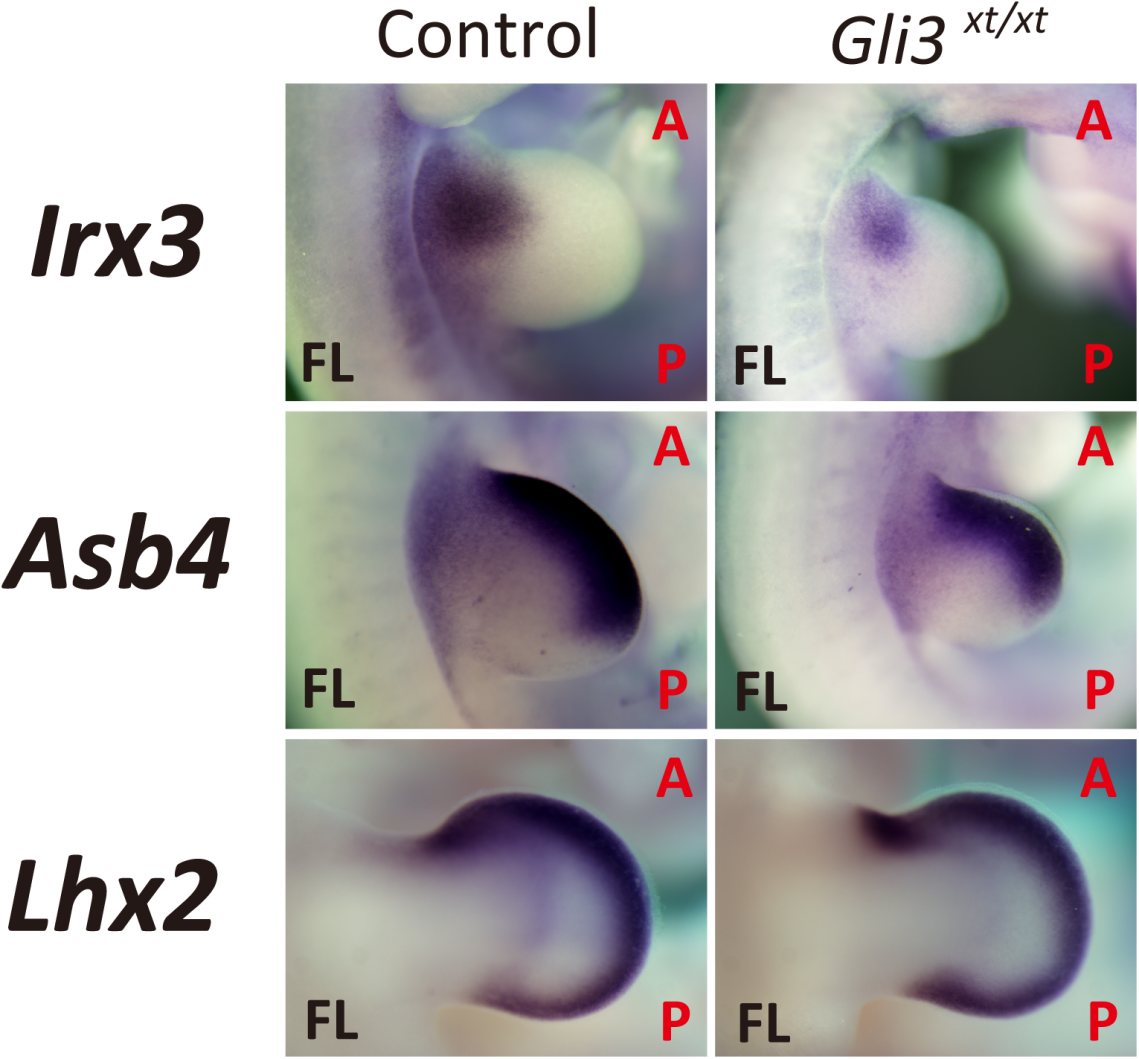
Reduced expression of *Irx3* in *Gli3*-deficient limb buds. Whole-mount *in situ* analysis of *Gli3*^*xt/xt*^ embryos (E10.5–12.5). Expression of *Irx3* was reduced while no significant change was seen in the expression of *Asb4* and *Lhx2* in *Gli3*-deficient limb buds. (Control: WT or Shh +/-; FL: Forelimb; HL: Hindlimb; A: Anterior; P: Posterior).

DIG-RNA probes were prepared as previously described [19] (Figs 1 and 2). *Shh*-KO and *Gli3*-KO embryos were described previously [20, 21] (Figs 3 and 4). WISH was performed according to standard procedures [19, 22]

### Plasmid construction

**Fig 5:**
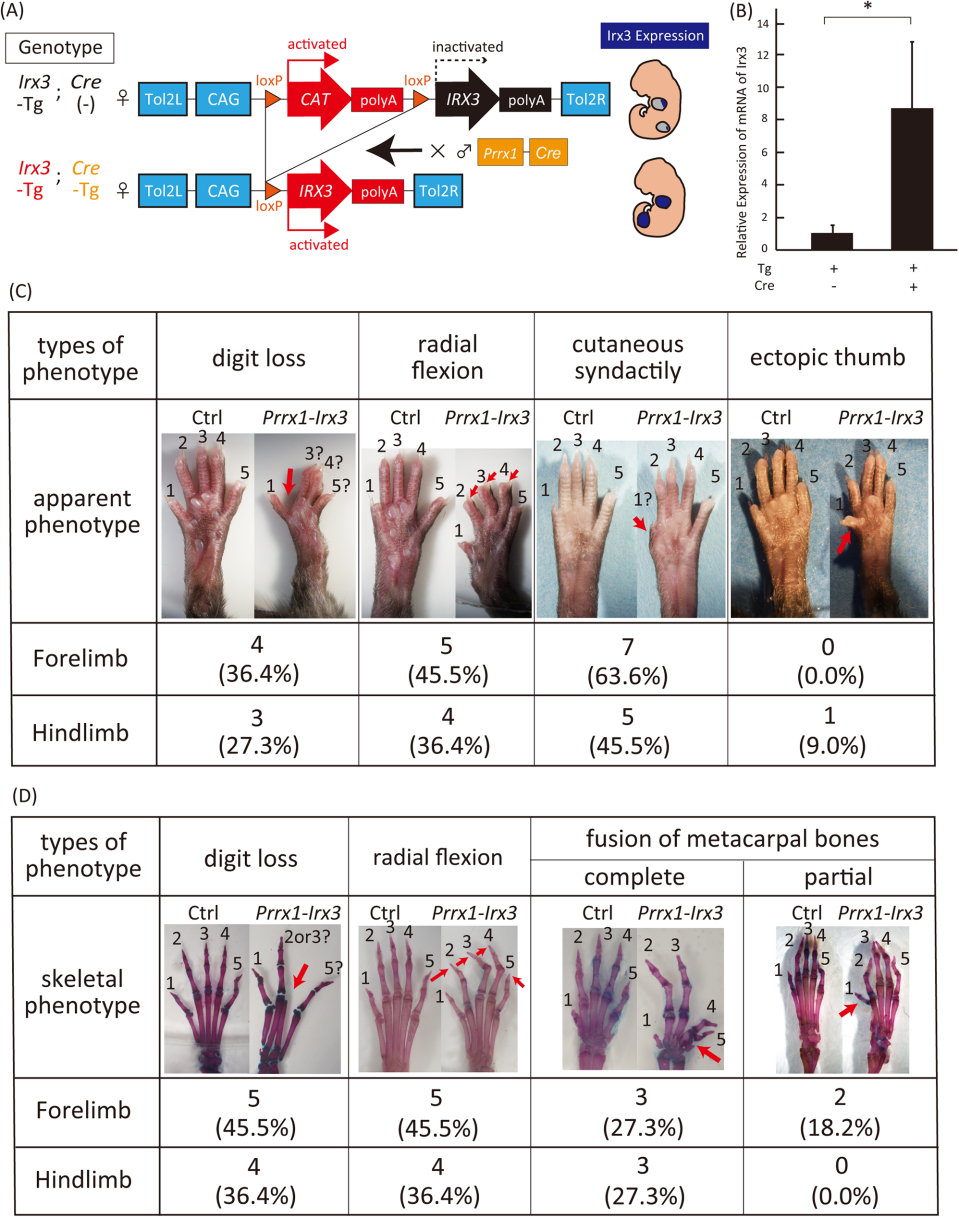
Generation and phenotypic analysis of transgenic mice (*Prrx1-Irx3)* overexpressing *Irx3* in the developing mouse limb bud. (A) Generation of transgenic mice overexpressing *Irx3* in the developing limb bud. (B) Eight fold enhanced expression of *Irx3* in the limb tissue of *Prrx1-Irx3* than the control (*Irx3*-Tg/*Cre* -). All data were expressed as the means ± SEM, (n = 5). *P < 0.05. (C-D) Different phenotypes of *Prrx1-Irx3* autopod were classified according to (C) apparent or (D) skeletal malformation (*Prrx1-Irx3*: *Irx3-Tg* and *Cre-Tg*; Ctrl: *Irx3-Tg* and *Cre (-)*).

The insert sequence, *CAG*-loxP-*CAT*-polyA-loxP-polyA, [23] was amplified by PCR. The linear vector from the pT2AL200R175-*CAGGS*-eGFP plasmid was amplified by inverse PCR [24]. PrimeSTAR Max DNA polymerase (Takara Bio) was used to amplify both fragments. The insert fragment was cloned into the linear vector to generate the pT2AL200-*CAG*-loxP-*CAT*-polyA-loxP-polyA-R175 (pT2A-*CAG*-loxP) plasmid using the GeneArt Seamless Cloning and Assembly kit (Thermo Fisher Scientific) according to the manufacturer’s protocol. The targeting vector (pT2A-*CAG*-loxP-*Irx3*) for microinjection was prepared as follows: the coding DNA sequence (CDS) of *Irx3* from the limb bud cDNA of an ICR mouse (a strain of albino mice) was amplified by PCR using the KAPA HiFi Ready Mix (KAPA BIOSYSTEMS) and cloned into the pT2A-*CAG*-loxP plasmid. The pT2A-*CAG*-loxP-*Irx3* vector carried a floxed *CAT* gene and *Irx3* CDS inserted between 200 bp and 175 bp of minimal *Tol2* elements, respectively (Fig 5A). The inserted *Irx3* sequence was validated by DNA sequencing. All primers used for PCR are shown in S1 Table.

### Generation of transgenic *Irx3* overexpressing mice

The Institutional Animal Care and Use Committee of Tokyo Medical and Dental University approved all animal experiments. The mice were sacrificed by cervical dislocation to alleviate suffering. *Tol2* mRNA was transcribed from linearized pCS-mT2TP [25] using the mMessage mMachine SP6 Transcription kit (Thermo Fisher Scientific). The resulting transcripts were purified by LiCl precipitation. To generate an *Irx3* transgenic mouse carrying the floxed CAT gene adjacent to the CDS of *Irx3* (Fig 5A), the pT2A-*CAG*-loxP-*Irx3* vector and *Tol2* mRNA were microinjected into pronuclear-stage frozen BDF1 mouse embryos (Ark Resource) (S1A and S1B Fig). The resulting chimeric offspring (F0 mice) were crossed with C57BL/6 mice. To confirm germ-line transmission, the resulting progeny mice (F1) were genotyped by PCR analysis (S1C Fig). The *Irx3* transgenic female mice were then crossed with *Prrx1-Cre* transgenic male mice (Jackson Laboratory) to generate *Prrx1-Irx3* mice (Fig 5A). Primers used for genotyping are shown in S1 Table.

### RNA isolation, reverse transcription, and quantitative real-time PCR

Total RNA was extracted from the limb epidermis of *Prrx1-Irx3* or *Irx3-Tg* mice using ISOGEN (Nippon Gene). The extracted RNA was used as a template for reverse transcription using SuperScript II Reverse Transcriptase (Thermo Fisher Scientific). The mRNA expression of *Irx3* was quantified by quantitative real-time PCR using Power SYBR Green PCR Master Mix (Thermo Fisher scientific) and were normalized to *Gapdh* mRNA levels (Fig 5B). Sequences of primers used for real-time PCR are shown in S1 Table.

### Preparations of skeletal specimen

After observing the apparent phenotype of 3-week-old *Prrx1-Irx3* or *Irx3*-Tg mice (Fig 5C), skeletal preparations were made and analyzed.

Skin and viscera were removed from the limbs of transgenic *Prrx1-Irx3* or *Irx3* mice. The specimens were then dehydrated in 100% ethanol for 120 h and degreased in acetone for 48 h. Subsequently, samples were stained with Alcian blue solution (7.5 g Alcian blue 8GX (Sigma) in 10 ml glacial acetic acid (Sigma) and 40 ml of 95% ethanol) at RT for 12 h. After washing with 95% ethanol for 10 min, the samples were treated with 2% KOH and stained with Alizarin red solution (7.5 mg Alizarin red S (Sigma) in 100 ml of 1% KOH solution). Specimens were washed with 20% glycerol in 1% KOH for 120 h and then with 20% glycerol in 20% ethanol for 12 h (Fig 5D).

### Statistical analysis

The two-tailed independent Student’s t-test was used to calculate all *P* values. Asterisks in figures indicate differences with statistical significance as follows: *P < 0.05.

## Results

### Initial EMBRYS-based screening for candidate genes involved in AP patterning based on spatial localizations and temporal expression changes of 691 transcription-associated factors in the mouse limb bud

Previously, we established a WISH database of transcription factors that are present in mouse embryos termed EMBRYS [19]. To identify factors involved in limb bud patterning, the expression pattern of each factor was analyzed. According to the database, transcripts of certain transcription-associated factors displayed characteristic spatial localization and temporal expression changes in different parts of the mouse embryo at E9.5–11.5. Initially, 691 transcription-associated factors expressed in the mouse limb bud were selected and classified into categories based on their spatial expression patterns [26] (Figs 1 and 2). To our interest, some of the expression patterns were polarized towards the anterior part of the limb bud, resulting in a pattern of expression reminiscent of *Gli3* expression (Fig 1). We predicted that these candidates might play a crucial role in specifying the future anterior-side of the mouse limb during development.

Formerly, *Irx3* was identified as a transcription factor involved in specifying the anterior side of the limb bud [26, 27]. It is expressed on the proximal side of the primordial limb bud before E9.5. By E10.5, it localizes to the anterior-proximal region and the future radial side of the zeugopod and stylopod (Fig 1).

The SOCS box superfamily protein Asb4 mediates vascular differentiation [28]. Like *Irx3*, *Asb4* is expressed in the anterior half of the distal part of the limb bud at E9.5. From E10.5 to 11.5, *Asb4* localizes towards the anterior side of the limb bud (Fig 1).

*Lhx2*, a vertebrate homologue of *apterous*, is expressed under the confined distal mesoderm beneath the AER. Through the initiation and maintenance of the AER, *Lhx2* regulates limb bud outgrowth in the chick [29, 30]. The expression pattern of *Lhx2* was similar to those of *Irx3* and *Asb4* at E9.5. However, at E10.5, *Lhx2* was also expressed at the posterior margin of the limb bud, which was consistent with previous reports (Fig 1).

*Nr4a2* is a member of the nuclear receptor family of intracellular transcription factors and is involved in neuronal differentiation [31, 32]. It was found to be expressed towards the anterior end at E10.5–11.5 (Fig 1).

*Hhex* is a homeobox gene involved in the formation of the heart, vascular system, forebrain, thyroid, and liver [33, 34]. Additionally, it is known to regulate the AP patterning in *Xenopus* and mice [35]. *Hhex* was expressed as a spot on the anterior side of the limb bud at E11.5 (Fig 1).

Transcription factors with median-localized expression patterns were also identified in the present study. *Odd-skipped related* genes, such as *Osr1* and *Osr2*, encode C2H2 zinc-finger transcription factors and are involved in the development of the embryonic heart, urogenital system [36], and the secondary palate [37]. *Osr1* and *Osr2* display dynamic expression patterns in the developing limb bud and are highly conserved between chicks and mice. *Osr1* and *Osr2* are partially co-expressed in the proximal limb bud but show mutually exclusive expression patterns in the distal autopod at E11.5–12.5 [38]. Present findings from EMBRYS indicate expression of *Osr1* in the posterior-proximal part of the limb bud and in adjacent flanks at E9.5–10.5. Furthermore, the expression of *Osr1* was distributed throughout the median mesenchyme of the autopod, prospective zeugopod, and stylopod at E11.5. In contrast, the expression of *Osr2* was localized to the center of the limb bud at E10.5, and a shift in expression pattern was seen to the medial mesenchyme of the autopod at E11.5 (Fig 2).

*Nr2f2* is a nuclear receptor subfamily member that plays a crucial role in skeletal muscle development during the limb bud outgrowth [39]. *Nr2f2* was expressed in multiple developing tissues, such as the proximal region of the limb bud at E10.5 and in the center of the autopod at E11.5 (Fig 2).

*Gbx2* is a homeobox gene required for the morphogenesis of the hindbrain [40]. Gbx2 was expressed along the posterior-middle part of the limb bud at E9.5–10.5 and was almost undetectable at E11.5 (Fig 2).

*Goosecoid* (*Gsc)* encodes a member of the bicoid subfamily of the paired homeobox family and plays a crucial role in craniofacial and rib cage development [41, 42]. As described previously [43], *Gsc* was expressed in the anterior-proximal part of the limb bud at E10.5 and was localized to the proximal region of the autopods extending posteriorly to the zeugopods and stylopods (Fig 2).

*Eomes*, also known as *Tbr2* or *Eomesodermin*, is a member of the T-box family of transcription factors. It is associated with neurogenesis, cardiogenesis, and tumor immune response [44–46]. *Eomes* was localized to a single spot in the center of the autopod at E11.5 (Fig 2). Previous study indicated localization of *Eomes* expression to the prospective digits at E14.5 in autopods [47] and to the metacarpal pre-cartilage condensation at E16.5 [48].

In conclusion, the results from EMBRYS enabled selection of genes showing specific AP polarity expression in the developing limb bud. Further, we aimed at identifying distinctive genes expressed anteriorly as candidates for regulating the anterior development of the limb bud.

### Candidate genes expressed towards the anterior side of the limb buds selected from EMBRYS showed posteriorly expanded expression pattern in *Shh-KO* limb buds

Interestingly, each selected candidate gene displayed different spatio-temporal expression patterns, suggesting their distinctive developmental roles in the specification of the anterior-side mesenchymal cells during limb bud development.

*Shh* is a crucial morphogen in the developing limb bud imparting posterior identity to the limb mesenchymal cells. Additionally, SHH in the ZPA mediates AP polarization [12]. Accordingly, *Shh*-*KO* mice have digit 1 and a shortened zeugopod [49]. In contrast, *Gli3*-deficient mouse models, such as the *extra-toes* (*Xt*) mice, exhibits symmetric polydactyly, the so-called mirror image digit duplication [21, 49, 50]. *Shh* expression is activated by *Hand2* and retinoic acid [51]. Expression of *Shh*, is kept restricted to the posterior margin of the limb bud by the mutual antagonism between *Gli3* and *Hand2* [52]. In the present study, the identified candidate factors were further screened based on their regulation by SHH and Gli3 signaling during the development of the mouse limb bud.

To narrow down the selected candidates regulating anterior specification identified from EMBRYS, we performed WISH analysis of E10.5–11.5 limb buds from *Shh-KO* mouse embryos. Deletion of *Shh* resulted in widened posterior expression pattern of the selected genes (Fig 3).

*Irx3* showed a posteriorly expanded expression pattern in *Shh*-deficient developing limb buds than those of the wild type, which was consistent with previous results [26], suggesting a loss of *Irx3* expressional polarity caused by the absence of *Shh*. Similar results were observed for other candidate genes. *Asb4* expression, which was originally localized to the anterior-half of the distal wild type limb bud, showed extended expression to the complete distal part and in addition was posteriorly expanded in the *Shh*-KO limb bud. *Lhx2*, which displayed a localized expression pattern along the lateral ridge in the wild type limb bud [29, 30], was expanded to the whole ridge in the *Shh*-KO limb bud. Furthermore, *Nr4a2* and *Hhex* were no longer asymmetrically expressed and their expression were posteriorly expanded in *Shh*-KO limb buds (Fig 3).

Thus, the expression pattern of the analyzed genes indicates that they are inhibited by *Shh* in the posterior side of the limb bud. They typically function at the anterior side of the limb bud and are independent of SHH signaling or negative regulation by SHH.

### Disruption of *Gli3* affects the expression pattern of the selected candidates in the developing limb bud

Evaluation of changed expression pattern by WISH analysis of the selected candidates using *Shh*-KO limb buds revealed that all the candidates that displayed an anterior-localized expression pattern in wild type limb buds (Fig 1) expanded posteriorly in *Shh*-deficient limb buds (Fig 3). These results probably suggest their function as regulators specifying the anterior side of the developing limb bud. To further analyze these candidates, WISH analysis in *Gli3*-KO (*extra-toes*: *Gli3*^*xt/xt*^) embryos was performed and the change in expression pattern compared to the wild type limb bud was evaluated. We hypothesized that candidates regulated by the Gli3 pathway would show reduced expression in *Gli3*-deficient mouse limb buds.

Among the candidates analyzed, the expression domain of *Irx3* was reduced and was localized more anterior-proximally in *Gli3*-deficient limb buds (Fig 4), which was consistent with previous results using *Gli3/Kif7* double KO limb buds [26]. No significant changes were detected in the expression of *Asb4* and *Lhx2* in *Gli3*-deficient limb buds (Fig 4).

Collectively, through an EMBRYS-based stepwise selection method, several transcription-associated factors were narrowed down to few specific ones. *Irx3* was selected for further analysis because of its crucial role in specifying the anterior mesenchymal cells in the limb bud.

### Generation of a transgenic mouse model overexpressing *Irx3* using the *Tol2* transposon system for *in vivo* phenotypic analysis

To validate the screening system, *in vivo* experiments were designed to analyze *Irx3* by generating a transgenic mice overexpressing *Irx3* induced by Cre recombinase under the control of the *Prrx1* regulatory element (*Prrx1-Irx3* mouse) (Fig 5A). Firstly, an *Irx3* transgenic mouse carrying a floxed CAT gene adjacent to the CDS of *Irx3* was created. Protocols for *Tol2*-transposon-mediated generation of transgenic animals were as previously described [24, 25, 53, 54]. *Tol2* mRNA and the *Tol2* targeting vector were co-injected to facilitate the integration of large DNA fragments into the genome of pronuclear stage embryos (S1 Fig). The resultant mice were crossed with *Prrx1-Cre* male mouse to obtain the *Prrx1-Irx3* mouse strain (Fig 5A, S1B and S1C Fig) [55]. The expression level of *Irx3* in the *Prrx1-Irx3* limbs was approximately 8-fold higher than in the wild type limbs (Fig 5B).

The *Prrx1-Irx3* mice showed diverse limb malformations probably because of the disruption of autopod patterning. These phenotypes were highly penetrant and were classified into four categories: cutaneous fusion of digits 2–3 (63.6%), loss of digits 2–4 (45.5%), radial flexion of digits 2–4 (45.5%), and fusion of the metacarpal and metatarsal bones (45.5%) (Fig 5C and 5D). Thus, the *Irx3* gain-of-function mice displayed complicated autopod phenotypes suggesting a significant role of *Irx3* in regulating limb bud patterning, especially in the autopod.

In summary, the present study established an EMBRYS-based stepwise screening system for developmentally crucial transcription-associated factors. Candidates for anterior regulators, such as *Irx3*, were efficiently and systematically selected through multiple screening steps. *In vivo* analysis using transgenic mouse overexpressing *Irx3* was performed to assess the screening system. Finally, to confirm the effectiveness and efficiency of the screening system, *Prrx1-Irx3* mice were evaluated for complex phenotypes in the autopod.

## Discussion

Morphogenesis of the developing limb bud is a highly reproducible model to study body plan formation. The process involves axis formation, patterning, outgrowth, and differentiation through interweaving gene interactions [1, 2]. Several transcriptional regulators play crucial roles in developmental and differential processes [6, 17, 18]. However, the exact mechanisms that orchestrate these processes remain to be elucidated [3].

Previously, we have established EMBRYS as a tool to identify novel transcription-associated factors that are essential for development [19]. In the present study, we established an EMBRYS-based stepwise screening system for searching candidate transcription-associated factors in a systematic and efficient manner. Firstly, we used EMBRYS to select candidates that exhibited a characteristic polarity along the AP axis. The expression pattern of several candidates showed clear localization along the anterior side of the developing limb bud mesenchyme similar to the expression pattern of *Gli3* (Fig 1). To narrow down candidate transcription-associated factors that may play a role in establishing AP polarity in the limb bud under the regulation of *Shh* or *Gli3*, WISH analysis was performed in *Shh-* and *Gli3-* deficient embryos. The candidates exhibited a posteriorly expanded expression pattern in *Shh*-deficient compared to wild type embryos (Fig 3). Additionally, expression of *Irx3* was reduced in the limb buds of *Gli3*-deficient embryos than in the wild type embryos (Fig 4), which was consistent with the previous studies [26, 27, 29, 30]. We therefore decided to focus on *Irx3* and its role as an anterior regulator in the developing limb bud [33, 34]. A mouse model overexpressing *Irx3* specifically in the limb bud was established for *in vivo* analysis of *Irx3* (Fig 5A). The *Prrx1-Irx3* mice displayed diverse malformations in the autopods due to defects in AP polarity (Fig 5C and 5D). Taken together, we validated an example of the EMBRYS-based stepwise screening system where candidate transcription-associated factors were efficiently selected, narrowed-down, and then analyzed *in vivo* to clarify their developmental role.

Initial screening identified five candidates showing anteriorly localized expression pattern in the developing limb bud (Fig 1). Previous reports indicate that these transcription-associated factors might play important roles during development.

*Irx* encodes a highly conserved three-amino acid-loop-extension (TALE) class homeoprotein at the N-terminus [56] and an IRO box at the C-terminus. *Iro* was first identified as a prepatterning gene for *Drosophila* bristles [57, 58]. *Iro* and *Irx* consist of clusters. In mammals, the *Irx* family consist of 6 paralogs that are classified into 2 clusters: *Irx1*, *2*, and *4*, and *Irx3*, *5*, and *6* are classified into the *IrxA* and *IrxB* clusters, respectively [59, 60]. The characteristic genomic structures of *Iro* and *Irx* are highly conserved among *Drosophila* [57, 58, 60], *Xenopus* [60–66], zebrafish [67], chicken, [68] and mammals [27, 64, 69–78]. Furthermore, transcripts in the same clusters show almost identical expression patterns [69, 76]. Previous studies have shown that they play crucial roles in the development of sensory organs [57, 58], the nervous system [60–63, 65, 67, 71, 76, 79], the retina [63, 71], kidneys [64, 66], the heart [68, 69, 75, 77], female gonads [74], and limb buds [26, 27, 69, 71, 78].

*Asb4* mediates oxygen-dependent vascular differentiation [28]. *Lhx2* regulates limb bud outgrowth through the initiation and maintenance of the AER in chick embryos [29, 30]. *Nr4a2* regulates the differentiation and maintenance of the dopaminergic system, [31, 32] and might also be relevant in patterning the forelimb bud based on its expression pattern [80]. *Hhex* is involved in the development of the heart, vascular system, forebrain, thyroid, and liver [33, 34]. To our interest, *Hhex* has been reported to regulate AP formation along the trunk in *Xenopus* and mice [35]. However, despite the characteristic expression pattern of the candidate genes in the developing limb bud, their role in patterning the limb bud along the AP axis has not been elucidated.

The initial screening of the candidate genes provided us with clues about their functions. Through EMBRYS and subsequent analysis of altered temporal expression, the list of candidate genes were further refined. *Gli3*, *Irx3*, *Asb4*, and *Lhx2* were predicted to be expressed in significantly wide regions in the E9.5 limb bud, suggesting their activated expression in the formation of the limb bud and their probable involvement in the early specification of the AP axis. In contrast, the expression of *Nr4a2* and *Hhex* were observed at a later stage of the developing limb bud, suggesting their involvement at a later stage of development such as late proliferation, differentiation, or digit specification.

Spatial expression patterns also suggested clues about the function of the candidates. At E11.5, *Irx3* was expressed along the radial side of the zeugopods and stylopods, suggesting its involvement in the outgrowth of these structures. *Asb4* was expressed in mesenchymal cells along the margin of the limb bud. Considering the previous reports [28], *Asb4* might be involved in angiogenesis along the anterior side of the developing limb bud, regulating proliferation and differentiation of anterior mesenchymal cells. From this viewpoint, *Lhx2* showed similar marginal expression patterns whereas *Nr4a2* and *Hhex* showed spotty expression patterns that were not marginal but were still included along the anterior side of the limb bud.

Following the initial screening, the regulation of candidate factors by SHH or Gli3 signaling during limb bud development was further analyzed. Each of these five candidates displayed a significant posterior expression expansion in the *Shh-KO* limb bud. Interestingly, these expansions resulted in uniform expression patterns along the AP axis, which might be attributed to the loss of AP polarity resulting from the lack of *Shh*. On the other hand, it was also impressive that not all of these candidates showed significant expression reductions in *Gli3*-deficient limb buds. This suggests that some of the candidates might be regulated by different functions of Gli3 under SHH signaling pathway during the anterior specification of the limb bud progenitor cells.

Of the candidates that were identified during this screen, *Irx3* was identified for further analysis. It has been implicated that the *IrxB* gene cluster, including *Irx3* and *5*, is involved in AP patterning of the developing limb bud [26, 27]. Previous study has indicated the regulation of *Gli3*, *Shh*, *Fgf8*, and their downstream target genes by *Irx3* resulting in the specification of anterior progenitors in the developing limb buds [27].

We cast a focus on *Irx3* as an example for *in vivo* analysis following the EMBRYS-based screening steps. Phenotypic analysis of *Prrx1-Irx3* mice displayed 4 types of autopod malformation: cutaneous fusion of interdigits, deficit of digits 2–4, radial deviation of digits 2–4, and fusion of the metacarpal and metatarsal bones (Fig 5C and 5D). Some of these phenotypes were observed in family members with mutations in *HOXD13* or *HOXA13* genes [81] and in *HOXD13-KO* mice [82]. In addition, *Prrx1-Hand2; Gli3* conditional double KO mice displayed similar phenotypes including the fusion of the metatarsals or a digit deficit [83]. These data suggests that overexpression of *Irx3* affects digit patterning in the developing limb bud. Overall, the present study demonstrates proof-of-concept for our screening system in identifying transcription-associated factors that regulate AP patterning in the limb.

Additional genes, such as *Gsc*, *Eomes*, and *Osr2* were clearly expressed in the middle region of the limb bud with a spotted pattern after E10.5 (Fig 2). Gsc is one of the transcription factor expressed in the Spemann’s organizer of the *Xenopus* embryo. When *Gsc* mRNA was microinjected into the ventral cells, twinned axes were induced, indicating that Gsc was sufficient to function like the Spemann's organizer [84]. *Eomes* is expressed in the mesoderm of Xenopus embryos, and ectopic expression of *Eomes* in the animal cap induces nearly all of the mesodermal genes [85]. *Osr2* is initially expressed in early gastrulation in the mesoderm and endodermal region and is necessary for kidney induction [86]. Since these key transcription factors of early gastrulation are also expressed adjacently in the limb mesenchyme after E10.5, it is interesting to speculate that a key transcription network interacting with the secreted growth factors during *Xenopus* gastrulation was co-opted in late limb mesenchyme induction.

In summary, we established an EMBRYS-based stepwise screening system to identify candidates for transcription-associated factors that might be involved in developmental processes such as AP patterning of the limb bud. This system is widely applicable to embryonic tissues other than the limb bud or to transcription-associated factors with other functions such as differentiation, proliferation, and pluripotency. The benefit of this screening system is the easy and efficient access to 3-dimensional and spatio-temporal gene expression patterns for each gene in an initial high-throughput screen. This enables quick and efficient analysis of expressional dynamics along transcriptional hierarches and to predict their roles during development. Moreover, following the initial screen, candidates of interest under the control of developmental master genes could be effectively selected. This is then followed by in vivo experiments, performed more efficiently nowadays because of improvements in genome editing technologies. As a result, we can gain insight into the spatio-temporal regulations of body plan formation by using the limb bud as a model to study the mechanisms of specification and morphogenesis.

## Supporting information

S1 Fig Efficient generation of *Irx3* transgenic mice via the *Tol 2* transposon system. (A) *Irx3* transgenic mice was generated using the *Tol2* transposon system. (B) High efficiency of *Irx3* transgenesis compared to a gene targeting system. (C) Genotyping strategy of transgenic *Irx3* mice.
(TIF)

S1 Table Primer sets used for plasmid preparation, real-time PCR, and genotyping. (XLSX)
