# Supplementary figures and images for: Analysis of transcription factors expressed at the anterior mouse limb bud
Source: PLoS One. 2017 May 3;12(5):e0175673. doi: 10.1371/journal.pone.0175673 (PMC5415108; doi:10.1371/journal.pone.0175673)

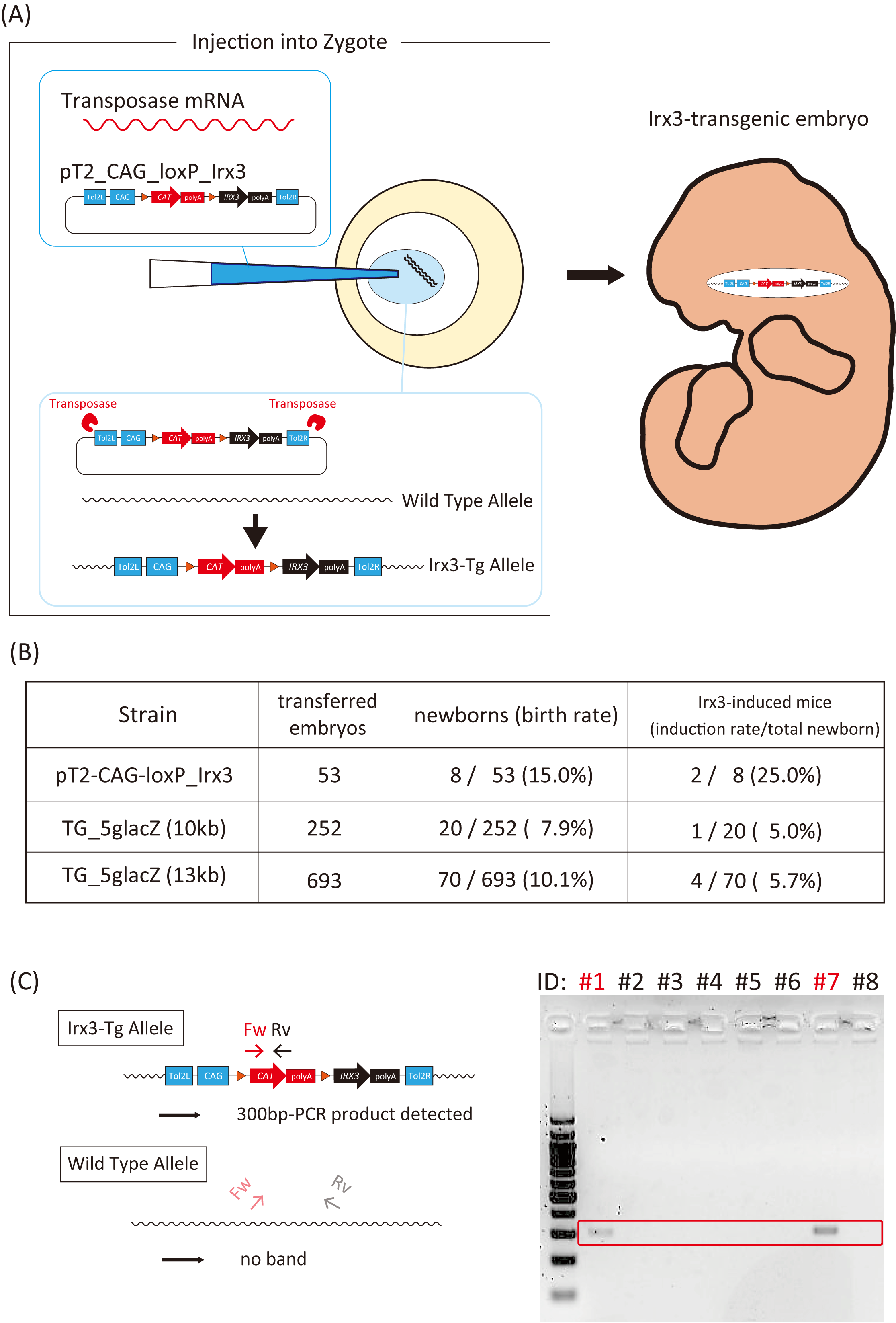

Supplement: S1 Fig — (A) Irx3 transgenic mice was generated using the Tol2 transposon system. (B) High efficiency of Irx3 transgenesis compared to a gene targeting system. (C) Genotyping strategy of transgenic Irx3 mice. (TIF) [file pone.0175673.s001.tif]
